# Spontaneous Facial Expressions and Micro-expressions Coding: From Brain to Face

**DOI:** 10.3389/fpsyg.2021.784834

**Published:** 2022-01-04

**Authors:** Zizhao Dong, Gang Wang, Shaoyuan Lu, Jingting Li, Wenjing Yan, Su-Jing Wang

**Affiliations:** ^1^Key Laboratory of Behavioral Science, Institute of Psychology, Chinese Academy of Sciences, Beijing, China; ^2^School of Computer Science, Jiangsu University of Science and Technology, Zhenjiang, China; ^3^Department of Psychology, University of the Chinese Academy of Sciences, Beijing, China; ^4^Department of Applied Psychology, College of Teacher Education, Wenzhou University, Zhejiang, China

**Keywords:** expressions, micro-expressions, action unit, coding, cerebral cortex, facial muscle

## Abstract

Facial expressions are a vital way for humans to show their perceived emotions. It is convenient for detecting and recognizing expressions or micro-expressions by annotating a lot of data in deep learning. However, the study of video-based expressions or micro-expressions requires that coders have professional knowledge and be familiar with action unit (AU) coding, leading to considerable difficulties. This paper aims to alleviate this situation. We deconstruct facial muscle movements from the motor cortex and systematically sort out the relationship among facial muscles, AU, and emotion to make more people understand coding from the basic principles:

1. We derived the relationship between AU and emotion based on a data-driven analysis of 5,000 images from the RAF-AU database, along with the experience of professional coders.
2. We discussed the complex facial motor cortical network system that generates facial movement properties, detailing the facial nucleus and the motor system associated with facial expressions.
3. The supporting physiological theory for AU labeling of emotions is obtained by adding facial muscle movements patterns.
4. We present the detailed process of emotion labeling and the detection and recognition of AU.

Based on the above research, the video's coding of spontaneous expressions and micro-expressions is concluded and prospected.

## 1. Introduction

Emotions are the experience of a person's attitude toward the satisfaction of objective things and are critical to an individual's mental health and social behavior. Emotions consist of three components: subjective experience, external performance, and physiological arousal. The external performance of emotions is often reflected by facial expression, which is an important tool for expressing and recognizing emotions (Ekman, 1993). Expressing and recognizing facial expressions are crucial skills for human social interaction. It has been demonstrated by much research that inferences of emotion fromfacial expressions are based on facial movement cues, i.e., muscle movements of the face (Wehrle et al., 2000).

Based on the knowledge of facial muscle movements, researchers usually described facial muscle movement objectively by creating facial coding systems, including Facial Action Coding System (FACS) (Friesen and Ekman, 1978), Face Animation Parameters (Pandzic and Forchheimer, 2003), Maximally Discriminative Facial Movement Coding System (Izard and Weiss, 1979), Monadic Phases Coding System (Izard et al., 1980), and The Facial Expression Coding System (Kring and Sloan, 1991). Depending upon the instantaneous changes in facial appearance produced by muscle activity, majority of these facial coding systems divide facial expressions into different action units (AUs), which can be used to perform quantitative analysis on facial expressions.

In addition to facial expression research based on psychology and physiology, artificial intelligence plays a vital role in affective computing. Notably, in recent years, with the rapid development of computer science and technology, the deep learning methods begin to be widely adopted to detect and recognize automatically by facial action units and makes automatic expression recognition possible in practical applications, including the field of security (Ji et al., 2006), clinical (Lucey et al., 2010), etc. The boom in expression recognition is attributed to many labeled expression datasets. For example, EmotioNet has a sample size of 950,000 (Fabian Benitez-Quiroz et al., 2016), which is large enough to fit the tens of millions of learned parameters in deep learning networks. The AU and emotion labels are the foundation for training the supervised deep learning networks and evaluating the algorithm performances. In addition, many algorithms are developed based on AU because of its importance (Niu et al., 2019; Wang et al., 2020).

However, the researchers found that ordinary facial expressions, i.e., macro-expressions, can not reflect a person's true emotions all the time. By contrast, the emergence of micro-expression has been considered as a significant clue to reveal the real emotion of humans. Studies have demonstrated that people would show micro-expressions in high-risk situations when they try to hide or suppress their genuine subjective feelings (Ekman and Rosenberg, 1997). Micro-expressions are brief, subtle, and involuntary facial expressions. Unlike macro-expression, micro-expression lasts only 1/25–1/5 s (Yan et al., 2013).

Micro-expression spotting and recognition have played a vital role in defense, suicide intervention, and criminal investigation. The AU-based study has also contributed to micro-expressions analysis. For instance, Davison et al. (2018) created an objective micro-expression classification system based on AU combinations; Xie et al. (2020) proposed an AU-assisted graph attention convolutional network for micro-expression recognition. Micro-expression has the characteristics of short duration and subtle movement amplitude, which causes that the manual annotation of ME videos requires the data processing personnel to view the video sample frame by frame slowly and attentively. Accordingly, long working hours increase the risk of errors. Furthermore, the current sample size of micro-expressions is still relatively small due to the difficulty of elicitation and annotation.

The prevailing annotation method is to annotate the AU according to the FACS proposed by Ekman et al. (Friesen and Ekman, 1978). FACS is the most widely used face coding system, and the manual is over 500 pages long. The manual covers Ekman's detailed explanation of each AU and its meaning, providing schematics and possible combinations of AUs. However, when AU is regarded as one of the criteria for classifying facial expressions (macro-expressions and micro-expressions), a FACS-certified expert is generally required to perform the annotation. The lengthy manual and the certification process have raised the barrier for AU coders.

Therefore, this paper focuses on macro-expression or micro-expression that responds to genuine emotions and analyzes the relationship between the cerebral cortex, which controls facial muscle movements, facial muscles, action units, and expressions. We theoretically deconstruct AU coding based on these analyses, systematically highlight the specific regions for each emotion. Finally, we provide an annotation framework for the annotator to facilitate the AU coding, expression labeling, and emotion classification.

This paper is an extended version of our ACM International Conference on Multimedia(ACM MM) paper (Zizhao et al., 2021), in which we make a brief guide to coding for spontaneous expressions and micro-expressions in video, and make the beginner to code get started as quickly as possible. In this paper, We discuss in further detail the principles of facial muscle movement from the brain to the face. Specifically, we show the cortical network system of facial muscle movement, introduce the neural pathways of the facial nucleus that control facial muscles, and the influence of other motor systems on the motor properties of the face. Secondly, we explain the relationship between AU and the six basic emotions with a physiological explanation. Finally, the coding of spontaneous expressions and micro-expressions is summarized in emotion label and AU detection and recognition research.

The following of this article is organized as follows: section 2 introduces the relationship between AU and emotions through the analysis of 5,000 images in RAF-AU database; section 3 demonstrates the nervous system of facial muscle movement; section 4 describes the muscles groups targeting the facial expression; section 5 exhibits the process of emotion labeling; section 6 shows detection and recognition research of AU; section 7 presents our conclusion and perspective on coding for spontaneous expressions and micro-expressions in videos.

## 2. Action Units and Emotions

**Figure 1:**
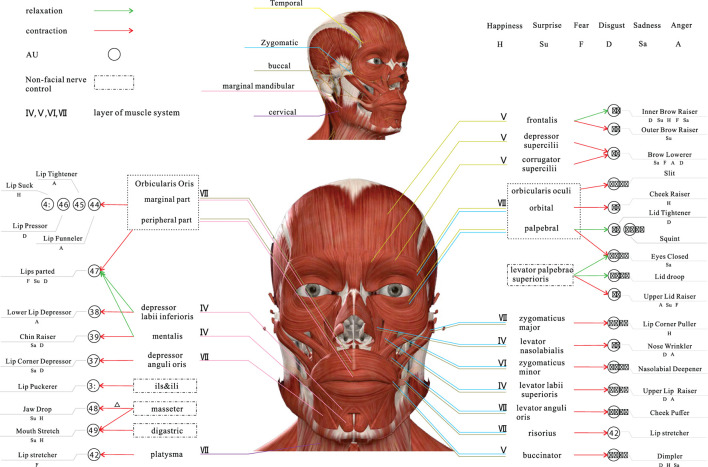
An overview of relationships of facial muscle, AU and emotion based on facial nerve (Zizhao et al., 2021).

Human muscle movements are innervated by nerves, and the majority of facial muscle movements are controlled by the seventh nerve in the brain, the facial nerve (Cranial Nerve VII, CN VII). The CN VII is divided into five branches, including the *temporal* branch, *zygomatic* branch, *buccal* branch, *marginal mandibular* branch and *cervical* branch (Drake et al., 2009). These branches are illustrated in the upper part of Figure 1.

The *temporal* branch of the CN VII is located in the upper and anterior part of the auricle and innervates the *frontalis, corrugator supercilii, depressor supercilii, orbicularis oculi*. The *zygomatic* branch of the CN VII begins at the *zygomatic bone* and ends at the lateral orbital angle, innervates the *orbicularis oculi* and *zygomaticus*. The *buccal* branch of the CN VII is located in the inferior box area and around the mouth and innervates the *Buccinator, orbicularis oris* and other orbicularis muscles. The *marginal mandibular* branch of the CN VII is distributed along the lower edge of the mandible and ends in the descending *depressor anguli oris*, which innervates the lower lip and chin muscles. The *cervical* branch of the CN VII is distributed in the cervical region and innervates the *platysma*.

All facial muscles are controlled by one or two terminal motor branches of the CN VII, as shown in Figure 1. One or more muscle movements can constitute AUs, and different combinations of AUs show a variety of expressions, which ultimately reflect human emotions. Therefore, it is a complex process from muscle movements to emotions. We conclude the relationship between AU and emotion based on the images in the RAF-AU database (Yan et al., 2020) and the experience of professional coders.

### 2.1. The Data-Driven Relationship Between AU and Emotion

All the data, nearly 5,000 images used to analyze, are from RAF-AU (Yan et al., 2020). The database consists of face images collected from social networks with varying covering, brightness, resolution, and annotated through human crowdsourcing. Six basic emotions and one neutral emotion were used in the samples. Crowdsourced annotation is a method, which may help sag facial expressions in a natural setting by allowing many observers to tag a target heuristically. Finally, the probability score that the picture belongs to a specific emotion is calculated. The database contains about 200,000 facial expressions labeling because that about 40 independent observers tagged each image. It should be noted that although the source image materials are diverse, the judging group of raters is relatively narrow because the taggers are all students.

The corresponding annotation contains both the expert's AU labels and the emotion score obtained from the crowdsourcer's label statistics for each image. We analyzed only the contribution of AUs to the six basic emotions with two methods. One method is to take the highest score as the emotion of the image and then combine it with the labeled AU. In this method, repeated combinations must be removed to avoid the effect on the results due to the predominance of one sample type, i.e., to mitigate the effect of sample imbalance. Another is to count the weighted sum of the contributions of all AUs to the six emotions without removing repetitions. The pseudocode details of these two methods are shown in Algorithms 1 and 2. Tables 1, 2 list the Top 10 AUs contributing to the six basic emotions, respectively. From Table 1, it can be seen that the contribution of AU25 is very high in the six basic emotions, which makes no sense because the movement of opening the corners of the mouth in AU25 is caused by the relaxation of the lower lip muscles, the relaxation of the genital muscles, and the orbicularis oris muscle. According to our subjective perception, AU25 rarely appears when we have three emotions: happiness, sadness, and anger. The abnormal top statistical data in Table 1 may be caused by the shortcomings of crowdsourced annotations, i.e., the subjective tendency or random labeling of some individuals.

**Algorithm 1:** 

1: Initialization: AU's contribution array to emotions *C*[6][*M*] = {0}
2: *M*: Max AU number, *N*: Number of samples, *i* = 0.
3: **repeat**
4: *i*←*i*+1
5: Split the AU combination into a single AU set
6: Take the maximum score of the six emotions as the emotion of the sample, defined as *E*
7: **if** the combination of AU and emotion *E* first appears **then**
8: Add the emotion score of the sample to the emotion AU
9: **end if**
10: **until** *i*>*N*
11: Calculate the proportion of AU in each emotion
12: Sort *C* in descending order
**Output:** Contribution array *C*

**Algorithm 2:** 

1: Initialization: AU's contribution array to emotions *C*[6][*M*] = {0}
2: *M*: Max AU number, *N*: Number of samples, *i* = 0.
3: **repeat**
4: *i*←*i*+1
5: Split the AU combination into a single AU set
6: Add the score of each emotion in the sample to *C*
7: **until** *i*>*N*
8: Sort *C* in descending order
**Output:** Contribution array *C*

**Table 1:** Top 10 of AU's contribution to the six basic emotions (Method 1).

**Emotion**	**AU**	**Score**	**AU**	**Score**	**AU**	**Score**	**AU**	**Score**	**AU**	**Score**	**AU**	**Score**	**AU**	**Score**	**AU**	**Score**	**AU**	**Score**	**AU**	**Score**
Happiness	25	0.1577	12	0.1424	10	0.0725	1	0.0649	6	0.0616	2	0.0565	26	0.0506	9	0.0498	4	0.0472	27	0.0447
Surprise	25	0.1760	1	0.1093	5	0.1088	2	0.0962	26	0.0772	12	0.0683	10	0.0499	27	0.0499	16	0.0473	4	0.0452
Anger	25	0.1454	10	0.1118	4	0.1034	9	0.0997	16	0.0701	12	0.0506	27	0.0502	5	0.0480	26	0.0402	7	0.0365
Fear	25	0.1669	12	0.0997	1	0.0873	27	0.0866	5	0.0835	4	0.0742	10	0.0734	16	0.0703	2	0.0580	26	0.0479
Disgust	4	0.1303	25	0.1226	10	0.1199	9	0.0782	17	0.0611	1	0.0488	12	0.0488	7	0.0470	26	0.0448	6	0.0398
Sadness	4	0.1526	25	0.1249	10	0.0745	1	0.0689	17	0.0578	12	0.0566	26	0.0505	15	0.0455	9	0.0455	16	0.0375

**Table 2:** Top 10 of AU's contribution to the six basic emotions (Method 2).

**Emotion**	**AU**	**Score**	**AU**	**Score**	**AU**	**Score**	**AU**	**Score**	**AU**	**Score**	**AU**	**Score**	**AU**	**Score**	**AU**	**Score**	**AU**	**Score**	**AU**	**Score**
Happiness	12	0.2312	35	0.2059	19	0.2015	2	0.1746	6	0.1635	28	0.1598	30	0.1543	27	0.1513	26	0.1470	14	0.1470
Surprise	2	0.3742	5	0.3625	35	0.3186	1	0.3051	26	0.2859	27	0.2832	21	0.2783	34	0.2721	28	0.2588	25	0.2398
Anger	9	0.3387	33	0.3333	16	0.2726	23	0.2662	7	0.2604	10	0.2549	30	0.2521	24	0.2346	29	0.2332	32	0.2328
Fear	20	0.2500	27	0.2054	33	0.1944	5	0.1854	16	0.1813	2	0.1674	1	0.1631	12	0.1444	30	0.1398	21	0.1307
Disgust	24	0.3112	32	0.3026	17	0.2942	15	0.2789	19	0.2642	14	0.2622	7	0.2484	4	0.2387	18	0.2377	9	0.2325
Sadness	39	0.4294	15	0.2810	43	0.2543	17	0.2355	4	0.1957	14	0.1686	6	0.1621	28	0.1598	7	0.1490	24	0.1467

However, there is room for improvement in the results obtained through the above data-driven approaches. The data-driven results can be affected by many aspects. Primarily, by the data source, such as the possible homogeneity of the RAF-AU database (number of subjects, gender, race, age, etc.), the uneven distribution of the samples, and the subjective labels based on human perception resulting from crowdsourcing annotation. Furthermore, the analysis method we used is based on a maximum value and probability weighting. Although straightforward, such analytical approaches represent the contribution of AU to the six basic emotions, are less comprehensive. More analysing methods are also needed in dealing with unbalanced data. In response to the challenges posed by data and analytical methods to data-driven methods, we could combine data-driven and experience-driven research methods. In this way, we could draw on the objectivity of data-driven and the robustness of experience-driven to realize the construction of the AU coding system for macro-expressions/micro-expressions.

### 2.2. The Experience-Driven Relationship Between AU and Emotion

There usually exists difficulties for the data-driven methods to analyze with theoretic basis. For example, the typical “black box” characteristic brings the problem of poor interpretability. Meanwhile, the results by data-driven are highly dependent on the quality (noiseless) and quantity (wide and massive) of the database. By comparison, the experience-driven method, based on the knowledge of coding and the common sense, is a way to label emotion. Three advantages are listed below: (1) The experience-driven method can help reduce the noise by using coding and common sense knowledge. (2) Experience-driven method has a reliable theory as a support, making the results convincing. (3) Experience-driven can often solve most universal laws with just a few simple formulas. Therefore, we combine experience-driven and data-driven methods to get the final AU and emotional relationship summary table, as shown in Table 3, by using their respective advantages.

**Table 3:** The relationship between AU and emotion.

**Emotion**	**AU**
Happiness	1, **6**, **12**, 14, 26, 27, **28**
Surprise	1, **2**, 5, 25, 26, 27
Anger	4, 5, 9, 10, **16**, **22**, **23**
Fear	1, 4, 5, **20**, 25
Disgust	1, 4, **7**, 9, 10, 14, 15, 17, **24**, 25
Sadness	1, 4, 14, 15, 17, **43**

*The bolded AU in the table indicates that the AU is only associated with the corresponding specific emotion, and not with other emotions*.

Specifically, firstly, based on the analysis results listed in Tables 1, 2 (data-driven), the preliminary selection is made by comparing the description and legend of each AU in FACS, and combining with the meaning of emotion. We obtained a preliminary AU system for emotion. Then, with large amounts of facial expression images on search engines such as Google and Baidu, the preliminary AU system for emotion was screened by eliminating non-compliant AU in these images. In this way, the ultimate relationship is shown in Figure 1 and Table 3.

Based on Table 3, we assume that the sets of six basic emotions containing AU are *S*_1_, *S*_2_, *S*_3_, *S*_4_, *S*_5_, and *S*_6_. Let *S* = {*S*_1_, *S*_2_, *S*_3_, *S*_4_, *S*_5_, *S*_6_}, then


(1) $$\begin{array}{l} Q_{i}=S_{i}\backslash {⋂}_{j}S_{j} \end{array}$$

where *i* = 1, ..., 6, and *j* = {1, ..., *i*−1, *i*+1, ..., 6}. ⋂ is the intersection operation of the set. \ represents the set of symmetric difference, for example, we assume that *A* = {3, 9, 14}, *B* = {1, 2, 3}, then *A*\*B* = {9, 14}. *Q*_*i*_ denotes the AU set that is exclusive to the *S*_*i*_ emotion.

According to Table 3, we can infer that the bloded AU is only associated with the corresponding specific emotion, and not with other emotions. See Table 3 in bold for details. Therefore, we can conclude that the appearance of certain AU represents related emotion. For instance, if AU20 appears, we assume that fearful emotion emerges.

## 3. Complex Cortical Networks of Facial Movement

**Figure 2:**
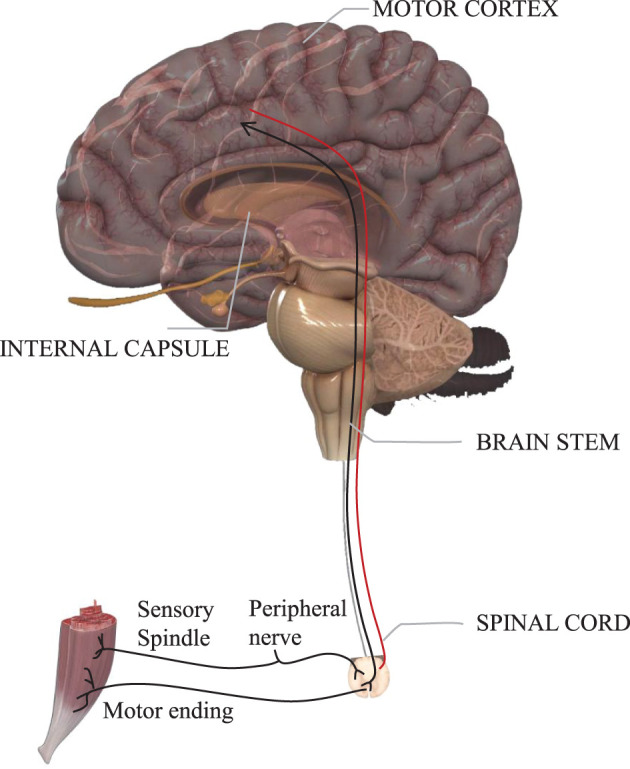
Motor neurons from the brain to the muscle.

The facial motor system is a complex network of specialized cortical areas dependent on multiple parallel systems, voluntary/involuntary motor systems, emotional systems, visual systems, etc., all of which are anatomically and functionally distinct and all of which ultimately reach the facial nucleus to govern facial movements (Cattaneo and Pavesi, 2014). The nerve that emanates from the facial nucleus is the facial nerve. The facial nerve originates in the brainstem, and its pathway is commonly divided into three parts: intra-cranial, intra-temporal, and extra-cranial (see Figure 2).

### 3.1. Facial Nucleus Controls Facial Movements

The human facial motor nucleus is the largest of all motor nuclei in the brainstem. It is divided into two parts: upper and lower. The upper part is innervated by the motor areas of the cerebral cortex bilaterally and sends motor fibers to innervate the muscles of the ipsilateral upper face; the lower part of the nucleus is innervated by the contralateral cerebral cortex only and sends motor fibers to innervate the muscles of the ipsilateral lower face. It contains around 10,000 neurons and consists mainly of the cell bodies of motor neurons (Sherwood, 2005).

A large number of neurons in the facial nucleus provides the anatomical basis for the various reflex responses of the facial muscles to different sensory modalities. For example, in the classic study by Penfield and Boldrey, it was found that the sensation of facial movement and the urge/desire to move the face was elicited by electrical stimulation of the cerebral cortex, causing movement of different parts of the face, as well as occurring in the absence of movement. Movements of the eyebrows and forehead were less frequent than those of the eyelids, and movements of the lips were the most frequent (Penfield and Boldrey, 1937).

Another way to assess the mechanism of inhibition within the cerebral cortex is to study the cortical resting period of transcranial magnetic stimulation. The cortical resting period is a period of inactivity called the silent period, when spontaneous muscle contraction is followed by a pause in myoelectric activity after the generation of motor evoked potentials by transcranial magnetic stimulation in the corresponding functional areas of the cerebral cortex. Studies on facial muscle movements have found that the silent period occurs after motor-evoked potentials in the pre-activated lower facial muscles (Curra et al., 2000), (Paradiso et al., 2005).

### 3.2. Cortical Systems Controls Facial Movement

The earliest studies on facial expressions date back to the nineteenth century. For example, the French neurophysiologist Duchenne de Boulogne (1806–1875) used electrical stimulation to study facial muscle activity (Duchenne, 1876). He used this experimental method to define for the first time expressions in different emotional states, including attention, relaxation, aggression, pain, happiness, sadness, cry, surprise, and fear, showing that each emotional state is expressed with specific facial muscle activity. Also, Duensing observed that there might be different neural structures involved between involuntary and emotional facial movements. Duensing's theory also influenced Charles Darwin's book *The expression of the emotions in man and animals* (1872) (Darwin, 1872).

Meanwhile, facial movements depend on multiple parallel systems that ultimately all reach the facial nucleus to govern facial movements. We focus on facial movements of expressions or micro-expressions, and two systems related to them have been discussed here: the voluntary/involuntary motor system and the emotional system.

#### 3.2.1. The Somatic Motor System

**Figure 3:**
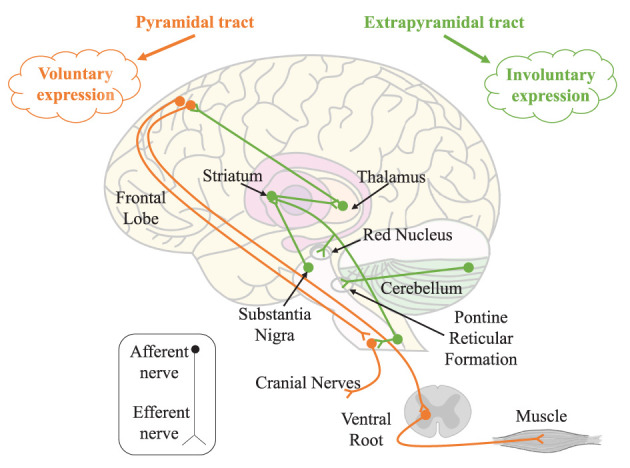
The somatic motor system. Voluntary and involuntary expressions are controlled by the pyramidal tract (orange trajectory) and extrapyramidal tract (green trajectory), respectively.

According to the form of movement of skeletal muscles, body movements are divided into voluntary and involuntary movements. Voluntary movements are emitted from the cortical centers of the brain and are movements executed according to one's consciousness, characterized by sensation followed by movement; involuntary movements are spontaneous movements that are not controlled by consciousness, such as chills. Meanwhile, the neuroanatomical distinction between voluntary and involuntary expressions has been established in clinical neurology (Matsumoto and Lee, 1993). Voluntary expressions are thought to emanate from the cortical motor tract and enter the facial nucleus through the pyramidal tract; involuntary expressions originate from innervation along the external pyramidal tract. See Figure 3.

Most facial muscles are overlapping, rarely contracting individually, and usually being brought together in synergy. In particular, these synergistic movements always occur during voluntary movements. For example, the *orbicularis oculi* and *zygomaticus* have a synergistic effect during the voluntary closure of the eyelid. In contrast, asymmetric movements of the face are usually thought to be the result of facial nerve palsy or involuntary movements (Devoize, 2011), for example, simultaneous contraction of the ipsilateral *frontalis* and *orbicularis oculi*, i.e., raising the eyebrows and closing the eyes at the same time. Babinski, a professor of neurology, considers that combined movements such as these cannot be activated by central mechanisms and cannot be replicated by volition. Therefore, facial asymmetry is always considered to be one of the characteristics of micro-expressions.

#### 3.2.2. The Emotional Motor Systems

Facial expressions are stereotyped physiological responses to specific emotional states, controlled by the voluntary and somatic systems controlled by the emotion-motor system (Holstege, 2002). Expression is only one of the somatic motor components of emotion, which also includes body posture and voice changes. However, in humans, facial expressions are external manifestations of emotions and are an essential part of human non-verbal communication (Müri, 2016), and a significant factor in the cognitive process of emotion. The emotion-motor pathway originates in the gray matter around the amygdaloid nucleus, lateral hypothalamus, and striatum. Most of these gray matter projects, in turn to the reticular formation to control facial premotor neurons, and a few project to facial motor neurons to control facial muscles directly.

In the study of traumatic facial palsy, a separation between the emotional motor system and the voluntary motor system at the brainstem level was found between facial movements (Bouras et al., 2007). It indicates that these two systems are entirely independent before the facial nucleus. This could be the reason why it is not possible to generate true emotional expression through volition. Therefore, emotion elicitation is required to produce behavioral (expression/micro-expression) responses through stimuli that induce emotion of the subject. It is relatively such expressions that have emotional significance. Moreover, there is also a strong correlation between the different activity patterns among facial muscles and the emotional valence of external stimuli (Dimberg, 1982). Similarly, the emotional motor system and the voluntary motor system interact and confront each other, and the results of this interaction are usually non-motor (e.g., motor dissonance) (Bentsianov and Blitzer, 2004).

Similar to the involuntary motor system, there is a small degree of asymmetry in the facial movements produced by the emotional motor system. However, the conclusions of this asymmetry are controversial. Many studies in brain-injured, emotionally disturbed, or normal subjects have shown that the majority of emotion expression, recognition, and related behavioral control is in the right hemisphere; that the right hemisphere dominates in the production of basic emotions, i.e., happiness and sadness, and the left hemisphere dominates in the production of socially conforming emotions, i.e., jealousy and complacency; and that the right hemisphere specializes in negative emotions while the left hemisphere specializes in positive emotions (Silberman and Weingartner, 1986).

## 4. The Specificity of the Relationship Between Facial Muscle and Emotions

According to Figure 1 and Table 3, we make further analysis of facial muscle and emotions to guarantee that each emotion can be targeted at a specific AU.

### 4.1. The Muscle That Classifies Positive and Negative Emotions

The basic dimensions for emotions are the two main categories, positive and negative emotions. Positive emotions are associated with the satisfaction of demand and are usually accompanied by a pleasurable subjective experience, which can enhance motivation and activity. By comparison, negative emotions represent a negative or aversive emotion such as sadness, disgust, etc., by an individual. The *zygomaticus* is controlled by the *zygomatic* branch of the CN VII. The *zygomatic* branch of the CN VII begins at the *zygomatic bone* and ends at the lateral orbital angle, innervates the *orbicularis oculi* and *zygomaticus*. The *zygomaticus* includes the *zygomaticus major* and the *zygomaticus minor*. The *zygomaticus major* begins in the *zygomatic bone*, and ends at the *angulus oris*. The responsibility of *zygomaticus* is to pull the corners of the mouth back or up to smile. The *zygomaticus minor* begins in the lateral profile of *zygomatic bone*, and ends at the *angulus oris*. The function is to raise the upper lip, such as grinning.

The *corrugator supercilii* begins in the medial end of the arch of the eyebrow and ends at the skin of the eyebrow, which is located at the *frontalis* and *orbicularis oculi* muscles back. It is innervated by the *temporal* branch of the CN VII. The contraction of *corrugator supercilii* depresses the brow and generates a vertical frown.

It has been found that the *corrugator supercilii* induced by unpleasant stimuli is more intense than that induced by pleasant stimuli, and the *zygomaticus* is more intense by pleasant stimuli (Brown and Schwartz, 1980). In a word, pleasant stimuli usually lead to greater electromyography(EMG) activity in the *zygomaticus*, whereas unpleasant stimuli lead to greater EMG activity in the frowning muscle (Larsen et al., 2003).

In the AU encoding process, *zygomaticus* activity and *corrugator supercilii* activity can reliably recognize positive emotion and negative emotion respectively. This conclusion also supports the discrete emotion theory (Cacioppo et al., 2000). For example, oblique lip-corner contraction (AU12), together with cheek raising (AU6) can reliably signals enjoyment (Ekman et al., 1990), while brow furrowing (AU4) tends to signal negative emotion (Brown and Schwartz, 1980). The correlation between emotion and facial muscle activity can be summarized as follows: (1) The main muscle area of the zygomatic is a reliable discriminating area for positive emotion; (2) The corrugator muscle area is a reliable identification area for negative emotion.

As shown in Figure 1, AU4, which is controlled by contraction of the depressor supercilii and corrugator supercilii, is present in all negative emotions. Most of the AU associated with happiness is controlled by the zygomatic branch, which mainly innervates the zygomatic muscle. Therefore, the coder should focus more on the cheekbones, i.e., the middle of the face and the mouth if they want to catch the expressions or micro-expressions elicited by positive stimuli. For those elicited by negative stimuli, the coder should focus more on the forehead, i.e., the eyebrows and the upper part of the face.

### 4.2. Further Specific Classification of the Muscles of Negative Emotions

In the six basic emotions, the negative emotions usually manifested as sadness, disgust, anger and fear, which are all highly associated with the *corrugator supercilii*, the brow and upper region. Therefore, in combination with the lower face, launching a further distinguishing of these four emotions from facial muscles is crucial for emotional classification.

#### 4.2.1. Muscle Group Specific for Sadness

The *depressor anguli oris* begins at the genital tubercle and the oblique line of the mandible, ends at the *angulus oris*. It is innervated by the *buccal* branch of the CN VII and the *marginal mandibular* branch. It serves to depress the *angulus oris*. The study found that when the participants produced happy or sad emotions by recalling, the facial EMG of the frowning muscle in the sadness was significantly higher than that in the happiness (Schwartz et al., 1976). This suggests that the combination of *corrugator supercilii* and textitdepressor anguli oris may be effective in classifying sad emotions.

#### 4.2.2. Muscle Group Specific for Fear

The *frontalis* begins in the *epicranial aponeurosis*, and extends to terminates in the skin of the brow and nasal root, and into the *orbicularis oculi* and *corrugator supercilii*. It is innervated by the *auricular posterior* nerve and the *temporal* branch of the CN VII. The *frontalis* is a vertical movement that serves to raise the eyebrows and increase the wrinkles at the level of the forehead, often seen in expressions of surprise. In expression coding, the action of raising the inner brow is coded as AU1. The *orbicularis oculi* begin in the pars nasalis ossis frontalis, the frontal eminence of the upper skeleton and the medial palpebral ligament, surrounds the orbit and ends at the adjacent muscles. Anatomically it is divided into the orbital and palpebral portions. It is innervated by the *temporal* and *zygomatic* branches of the CN VII. The function is to close the eyelid. In the study of the positive intersection of facial expressions and emotional stimuli, the researchers asked the subjects to maintain the fear feature of facial muscles, involving *corrugator supercilii, frontalis, orbicularis oculi*, and *depressor anguli oris* (Tourangeau and Ellsworth, 1979).

### 4.3. Distinguish the Special Muscle of Surprise

Surprise is an emotion that is independent of positive and negative emotions. For example, pleasant surprise, shock, etc., fall within the category of surprise. The study of people's surprise emotion has been started since Darwin (Darwin, 1872), and it is ubiquitous in social life and belongs to one of the basic emotions. Moreover, surprise can be easily induced in the laboratory.

Landis conducted the earliest study of surprising expressions (Landis, 1924). About 30% of people raised their eyebrows, and about 20% of people's eyes widened when a firecracker landed on the back of the subject's chair. Moreover, in discussing the evidence for a strong dissociation between emotion and facial expression, the research measured facial movements associated with surprise twice (see experiments 7 and 8). When subjects experienced surprise, the facial movements were described as frowning, eye-widening, and eyebrow raising (Reisenzein et al., 2006). Also, in exploring the distinction in dynamics between genuine and deliberate expressions of surprise, it was found that all expressions of surprise consisted mainly of raised eyebrows and eyelid movements (Namba et al., 2021). The facial muscles involved in these movements were: *corrugator supercilii, orbicularis oculi*, and *frontalis*. Details are described in section 4.2. The AUs associated with these facial muscles and movements include AU2, AU4, and AU5. As shown in Figure 1 and Table 3.

## 5. Emotion Label

Expressions are generally divided into six basic emotions, happiness, disgust, sadness, fear, anger and surprise. Micro-expressions are usually useful when there is a small negative micro-expression in a positive expression, such as “nasty-nice.” For micro-expressions, therefore, they are usually divided into four types, positive, negative, surprise and other. To be specific, positive expression includes happy expressions, which is relatively easy to be induced because of some obvious characteristics. Negative expressions like disgust, sadness, fear, anger, etc., are relatively difficult to distinguish, but they are significantly different from positive expressions. Meanwhile, surprise, which expresses unexpected emotions that can only be interpreted according to the context, has no direct relationship with positive or negative expressions. The additional category, “Others,” indicates expressions or micro-expressions that have ambiguous emotional meanings can be classified into the six basic emotions.

Emotion labeling requires the consideration of the components of emotions. Generally speaking, we need to take three conditions into account for the emotional facial action: AU label, elicitation material, and the subject's self-report of this video. Meanwhile, the influence of some habitual behaviors should be eliminated, such as frown when blinking or sniffing.

### 5.1. AU Label

For AU annotation, the annotator needs to be skilled in the facial coding system and watches the videos containing facial expression frame by frame. The three crucial frames for AU are the start frame (onset), peak frame (apex), and end frame (offset). Then we can get the expression time period for labeling AU. The start frame represents the time where the face changes from neutral expression. The peak frame is the time with the greatest extent of that facial expression. The end frame is the time where the expression ends and returns to neutral expression.

### 5.2. Elicitation Material

Spontaneous expressions have high ecological validity compared to posed expressions and are usually elicited with elicitation material. In psychology, researchers usually use different emotional stimuli to induce emotions with different properties and intensities. A stimulus is an important tool for inducing experimental emotions. We use stimuli materials, usually from existing emotional materials databases, to elicit different types of emotions of the subject.

### 5.3. Subject's Self-Report of This Video

After watching the video, the subjects need to evaluate the video according to their subjective feelings. This self-report is an effective means of testing whether emotions have been successfully elicited.

### 5.4. Reliability of Label

In order to ensure the validity or reliability of data annotation, the process of emotion labeling usually requires the participation of two coders and the calculation of inter-coders confidence must exist in a proper range. The formula is as follows 2:


(2) $$\begin{array}{l} R=\frac{N\times |{⋂}_{i=1}^{N}C_{i}|}{\left|{⋃}_{i=1}^{N}C_{i}\right|} \end{array}$$

where *C*_*i*_ represents the set of labeled emotions in the facial expression images by coder i(2 ≤ i ≤ N), respectively, and ∣·∣ represents the number of labeled emotion in the set after the intersection or merge operations.

The reason is that in the process of annotation, the coders must make subjective judgments based on their expertise. In order to make these subjective judgments as similar as possible to the perceptions of the majority of people, inter-rater reliability is of paramount importance. Inter-rater reliability is a necessary step for the validity of content analysis (emotion labeling) research. The conclusions of data annotation are questionable or even meaningless without this step.

It is mentioned above show that emotion labeling is a complex process, which needs coders to have the expertise with both psychology and statistics, increasing the threshold for being a coder. So we tried to find a direct relationship between emotions and facial movements to identify specific regions of emotions, as shown in Figure 1.

## 6. Detection and Recognition of AU

Facial muscles possess complex muscle patterns. Researchers have developed facial motion coding systems, video recordings, electromyography, and other methods to study and analyze facial muscle contractions.

The FACS coding system developed by Friesen and Ekman (1978) is based on the anatomical structure of facial muscles and is composed of all visible facial motion units AU under different intensities. So far, more than 7,000 AU combinations have been found in a large number of expressions. However, even for FACS coders, such labeling is time-consuming and labor-intensive.

Since then, the researchers have made some automatic coding attempts. For example, by analyzing the images in the video, it can automatically detect, track and classify the AU or AU combination that causes facial expressions (Lien et al., 2000). Nevertheless, unfortunately, image quality is especially susceptible to illumination, which, to some extent, limits such visible spectrum imaging technology.

To surmount such problem, researchers used facial electromyography, which is widely used in clinical research, to record AU muscle electrical activity (even visually imperceptible). This technique is susceptible to measuring the dynamics and strength of muscle contractions (Delplanque et al., 2009). However, there still exist some shortcomings: objective factors such as electrode size and position, epidermal cleanliness, and muscle movement methods, may interfere with the accuracy of the final experimental results and cause deviations in experimental conclusions. What's more, the number of muscles related to AU should theoretically be as much as that of electrodes, which also makes EMG a severe limitation as a non-invasive method.

Additionally, thermal imaging technology has also been applied to the study of facial muscle contraction and AU. Research has demonstrated that muscle contraction can cause skin temperature to increase (González-Alonso et al., 2000). For this reason, Jarlier et al. took thermal imaging as a tool to investigate specific facial heat patterns associated with the production of facial AUs (Jarlier et al., 2011). Therefore, thermal images can be used to detect and evaluate specific facial muscle thermal patterns (the speed and intensity of muscle contraction). Furthermore, this method avoids the lighting problems encountered when using traditional cameras and the influence of electrodes when using EMG.

## 7. Conclusion

In this article, with the help of statistical analysis, a data-driven approach is used to obtain a quantifiable system between AU and emotion. And then, we further obtain a robust correspondence system between AU and emotion by combining with an empirically driven comparison to actual data (from the web). In the next part, we introduce the cortical system that controls facial movements. Moreover, the physiological theoretical support for AU labeling of emotions was obtained by adding facial muscle movements. Finally, we sort out the process of emotion label and the research of AU recognition and detection. The main manifestations are listed below:

Based on the Figure 1 and Table 3, the theories of sections 3 and 4, we sum up the main points of coding in the article:

1. When corners of lips pulled up (AU12) appears, it can be coded as a positive emotion, i.e., happy; In addition, cheek rise (AU6), lip suck (AU28) are both happy specific action units and can also be coded as positive emotions;
2. When brow rise (AU2) is present, it can be coded as surprise;
3. When frown (AU4) is present, it can be coded as a negative emotion;
4. It can be coded as anger when gnashing (AU16, AU22 or AU23), which only occur in the specific action units, appear;
5. When movements of the eyebrows (AU1 and AU4), eyes (AU5) and mouth (AU25) are present simultaneously, they can be coded as fear;
6. It can be coded as disgusted when the specific action unit of disgust, lower eyelid rise (AU7), mouth tightly closed (AU24), is present;
7. It can be coded as sad when frown (AU4) and eyes wide open (AU5) are present at the same time; eyes closed (AU43) is the specific action unit for sadness and can also be coded as sadness.

## Data Availability Statement

Publicly available datasets were analyzed in this study. This data can be found here: http://whdeng.cn/RAF/model3.html#:$~$:text=a%20Real-world%20Affective%20Faces%20Action%20Unit%20%28RAF-AU%29database%20that,to%20annotating%20blended%20facial%20expressions%20in%20the%20wild.

## Author Contributions

ZD has contributed the main body of text and the main ideas. GW was responsible for empirical data analysis. SL was responsible for constructing the partial research framework. JL has contributed to the construction of the text and refinement of ideas and provided extensive feedback and commentary. S-JW led the project and acquired the funding support. All authors contributed to the article and approved the submitted version.

## Funding

This work was supported by grants from National Natural Science Foundation of China (61772511, U19B2032), National Key Research and Development Program (2017YFC0822502), and China Postdoctoral Science Foundation (2020M680738).

## Conflict of Interest

The authors declare that the research was conducted in the absence of any commercial or financial relationships that could be construed as a potential conflict of interest.

## Publisher's Note

All claims expressed in this article are solely those of the authors and do not necessarily represent those of their affiliated organizations, or those of the publisher, the editors and the reviewers. Any product that may be evaluated in this article, or claim that may be made by its manufacturer, is not guaranteed or endorsed by the publisher.
